# Effects of vegetation restoration on soil fungi community structure and assembly process in a semiarid alpine mining region

**DOI:** 10.3389/fpls.2025.1579142

**Published:** 2025-07-04

**Authors:** Yuanyuan Xue, Wei Liu, Qi Feng, Meng Zhu, Jutao Zhang, Lingge Wang, Zexia Chen, Xuejiao Li

**Affiliations:** ^1^ Key Laboratory of Ecological Safety and Sustainable Development in Arid Lands, Northwest Institute of Eco-Environment and Resources, Chinese Academy of Sciences, Lanzhou, China; ^2^ College of Resources and Environment, University of Chinese Academy of Sciences, Beijing, China

**Keywords:** vegetation restoration, soil fungal community, β diversity, community assembly mechanisms, alpine mining regions

## Abstract

**Introduction:**

Understanding responses of soil fungal community characteristics to vegetation restoration is essential for optimizing artificial restoration strategies in alpine mining ecosystems. Despite its ecological significance, current comprehension regarding the structure composition and assembly mechanisms of soil fungal communities following vegetation restoration in these fragile ecosystems remains insufficient.

**Methods:**

We used the high-throughput sequencing and null model analysis to determine the variations and environmental drivers of soil fungal community structures and assembly processes across different restoration chronosequences (natural plant sites, unrestored sites, 2-year restoration sites, and 6-year restoration sites) in a semiarid alpine coal mining region.

**Results:**

Artificial vegetation restoration significantly enhanced the α diversity of soil fungal communities while reducing β diversity. However, with prolonged restoration duration, we observed a significant decrease in α diversity accompanied by a corresponding increase in β diversity. Moreover, artificial restoration induced substantial modifications in soil fungal community composition. Taxonomic analysis demonstrated a distinct shift in dominant specialist species from Ascomycota in unrestored, natural plant, and 2-year restoration sites to Glomeromycota in 6-year restoration sites. Dispersal limitation and homogeneity selection were the predominant mechanism governing soil fungal community assembly, with its relative contributions varying significantly across restoration stages. In natural plant communities and unrestored sites, the structure of soil fungal community was primarily governed by dispersal limitation. The 2-year restoration sites exhibited a marked transition, with homogeneous selection emerging as the dominant assembly process, primarily influenced by soil sand content, total phosphorus (TP), total potassium (TK), and belowground biomass (BGB). This transition was accompanied by a significant reduction in the contribution of dispersal limitation.

**Discussion:**

As restoration progressed, the importance of homogeneous selection gradually decreased, while dispersal limitation regained prominence, with community structure being predominantly regulated by soil clay content, soil moisture content (SMC), and TP. Our results underscore the critical role of soil texture and phosphorus availability in shaping soil fungal community dynamics throughout the revegetation process.

## 1 Introduction

Coal mining, while contributing to economic development, causes a series of ecological disruptions, such as soil moisture loss, nutrient depletion, plant mortality, and shifts in soil microbial community structure and composition (de Quadros et al., 2016; Chen et al., 2020; Yuan et al., 2021). Numerous studies have found that vegetation restoration can effectively enhance soil nutrient content, improve soil quality, prevent soil erosion, and increase biodiversity, making it one of the most effective measures for mitigating ecological disruptions resulting from mining activities (Dao et al., 2023). A study conducted in the Moa mining area found that soil aggregate stability increased by 66% after planting *Casuarina equisetifolia* (Izquierdo et al., 2005). Dao et al. (2023) observed that during the vegetation recovery process over 5 to 14 years, the composition of soil microbial communities approached that of undisturbed soils. Additionally, Roy et al. (2020) demonstrated that appropriate vegetation interventions on coal mine wastelands, combined with fine-tuned nitrogen and phosphorus fertilizer doses, enabled the optimal growth of sandbuckthorn, thereby promoting and accelerating the ecological restoration of degraded coal mining ecosystems. Thus, the implementation of artificial measures can effectively promote ecological restoration and the recovery of ecosystem functions in mining areas (Chen et al., 2020).

The Muli coal mining area in the Qilian Mountains has abundant coal reserves, and it is not only the source region of the Datong River, but also an important regional water source and ecological safety barrier, occupying a crucial ecological position (Yang et al., 2018; Li et al., 2021). However, the ecosystem of the Muli coal mining area is highly vulnerable due to the cold climate, high altitude, and thin air. Meanwhile, coal mining activities have exacerbated ecosystem degradation, especially in permafrost environments, which will induce a series of problems (Ahirwal and Maiti, 2017). For example, open-pit mining directly removes the surface cover layer, damaging the insulating protective structure of the permafrost layer, accelerating its thawing, and disrupting the thermal balance of the permafrost. This leads to geological hazards such as frost heave and thaw subsidence, which in turn causes habitat degradation for plants and animals. Additionally, the large amount of organic carbon stored in the permafrost is released as CO_2_ through microbial decomposition upon thawing, further accelerating global warming (Ji et al., 2022). Thus, the natural restoration process is slow and difficult, and artificial interventions have become essential in the ecological restoration process of mining areas (Luo et al., 2020). Restoration duration is supposed to significantly affect vegetation and soil properties. The research conducted on the Tibetan Plateau by Yuan et al. (2021) indicated that extending the restoration period can significantly promote the accumulation of organic matter and improve soil structure. Additionally, Ma et al. (2019) found that typical vegetation restoration in mining areas positively affected soil quality improvement, while artificial maintenance should be strengthened to prevent soil quality degradation in future ecological management. Furthermore, researchers have observed that with increasing restoration time, the dominance of fungi significantly rises, such as Ascomycota and Zygomycota (Qiang et al., 2020).

As a vital member of the soil ecosystem, soil fungi are essential for nutrient cycling and soil structure development through aggregate formation, while also establishing symbiotic associations with plants to enhance their growth and development (Courty et al., 2010). Researches have demonstrated that Glomeromycota fungi enhances carbon sequestration and promote soil aggregation by producing glomalin-related soil proteins (Agnihotri et al., 2022; Wang et al., 2022). Ascomycota can produce various antimicrobial agents that protect plants from pathogen attack (Jibola-Shittu et al., 2024). And arbuscular mycorrhizal fungi not only supply essential mineral elements to host plants but also enhance resistance to pests, diseases, and abiotic stresses, thereby promoting plant health (Zhang X, et al., 2022). In addition, soil fungi was also used to indicate soil environment changes affected by environmental disturbances. For instance, the relative abundance of Glomeromycota is sensitive to salinity (Krishnamoorthy et al., 2014), while the relative abundance of Mortierellomycota can predict plant growth traits (Zhang et al., 2023). Mycorrhizal fungi have been identified as important predictors of global soil carbon storage (Averill et al., 2014). Diversity is one of the key characteristics for understanding and managing ecosystem functions, as well as evaluating the ecosystem’s resilience to disturbances. Generally, higher diversity enhances community stability and increases its ability to resist external disturbances (Liu et al., 2020). Therefore, an increasing number of studies emphasize the necessity of investigating the complete characteristics of fungal communities, including their diversity, structure, assembly, and driving factors.

Presently, most research has primarily focused on the effects of soil properties and plant communities on fungal communities following artificial restoration. In fact, the relative contributions of stochastic and deterministic processes vary depending on ecological and environmental conditions, while the impact of these processes following artificial restoration were less understood (Myers et al., 2013; Tripathi et al., 2018). After revegetation, the heterogeneity of fungal habitats increased, resulting more complicated assembly processes in fungal communities. Understanding the assembly processes of soil fungal communities under different restoration durations could provide scientific guidance for improving restoration outcomes in alpine mining areas. Especially in open-pit mining, the removal of surface soil leads to ecosystem degradation, resulting in reduced moisture retention capacity and soil organic matter, and the loss of biodiversity (Chen et al., 2020). Furthermore, plant growth and animal reproduction are inhibited by low temperatures and low oxygen levels in alpine mining areas, which significantly decrease plant diversity and soil nutrients (Ganesh et al., 2020). Therefore, we hypothesize that the soil fungal community diversity in unrestored sites of alpine mining areas is relatively low, with community assembly primarily driven by dispersal limitation. Following artificial restoration, increases in vegetation diversity and soil nutrient content promote the diversity of soil fungal community, with community assembly being mainly governed by deterministic processes.

Therefore, the main goals of this study were (1) to explore changes in the composition and diversity of soil fungal communities, and (2) to determine soil fungal community assembly process and dominant driving factors across different restoration chronosequences in alpine mining coalfields.

## 2 Materials and methods

### 2.1 Study area

**Figure 1:**
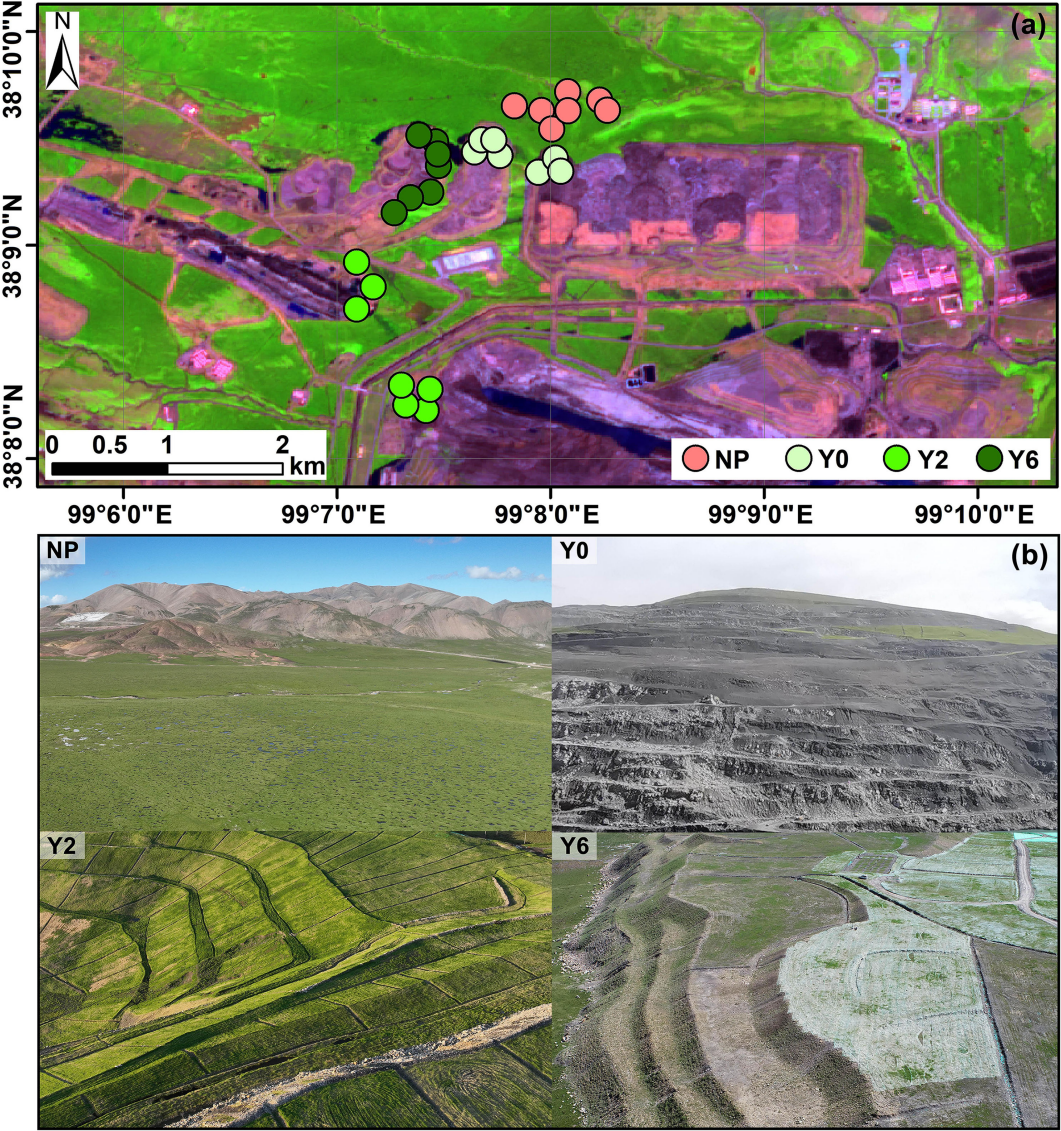
Location of sampling points in the Muli coalfield **(a)**, and the landscape of natural plant sites (NP), unrestored sites (Y0), 2-year restoration sites (Y2), and 6-year restoration sites (Y6) in study area **(b)**.

The study area is situated in the Muli coalfields (99°05′E–99°27′E, 38°05′N–38°27′N) of the middle Qilian Mountains, northwestern China (**Figure 1**). It lies in the northeastern part of the Qinghai-Tibet Plateau with an average elevation of 4100 m.a.s.l. The climate is typical semiarid alpine climate, with mean annual temperature, precipitation, and evaporation reaching to -4°C, 477 mm, and 1050 mm, respectively. The annual sunshine hours is 2942 h. The region is characterized by glacial landforms at the edge of the plateau, where permafrost (5 to 50 meters) and seasonal frost (1 to 3 meters) are extensively distributed (Bai, 2022). The predominant vegetation types include alpine meadows and alpine wetlands, with the dominant species being *Carex moorcroftii* and *Koeleria tibetica*, and the vegetation coverage is approximately 80%. The main soil type is classified as haplic leptosols according to the World Reference Base for Soil Resources, which is roughly referred to as alpine meadow soils according to the Chinese Soil Genetic Classification.

In the 1960s, small-scale mining activities started gradually, with large-scale extraction beginning in the early 21st century predominantly through open-pit mining with extensive excavation. To mitigating ecological disruptions resulting from mining activities, the government implemented comprehensive ecological remediation initiatives in the mining area between 2014 and 2018. However, persistent illegal coal extraction by certain enterprises led to renewed media exposure of unauthorized mining activities in 2020, generating substantial societal repercussions. In prompt response, governments launched extensive ecological restoration initiatives to rehabilitate the affected areas.

### 2.2 Plant and soil sample collection and analysis

Soil sampling was conducted in the Muli coalfield in August 2023. We selected soil sampling sites according to the restoration year, i.e., the landscape of natural plant sites (NP), unrestored sites (Y0), 2-year restoration sites (Y2), and 6-year restoration sites (Y6), with a total of 28 soil samples (7 replicates) from 0–20 cm were obtained. We measured Aboveground biomass (AGB), belowground biomass (BGB), litter biomass (Litter), bulk density (BD), soil moisture content (SMC), soil electrical conductivity (EC), pH, soil organic carbon (SOC), total nitrogen (TN), total carbon (TC), total potassium (TK), and phosphorus (TP). The fungal ITS-1 region was amplified with the primer pair ITS1 (5’-CTTGGTCATTTAGAGGAAGTAA-3’) and ITS2 (5’-GCTGCGTTCTTCATCGA TGC-3’) (Blaalid et al., 2013) to obtain the information of fungal community. More detailed descriptions of sampling protocols and procedures for laboratory analyses can be found in Xue et al. (2025). The raw sequencing reads were deposited in the NCBI database under the accession number PRJNA1208394.

### 2.3 Statistical analysis

The ANOVA and LSD tests were performed to analyze differences among groups using the ‘agricolae’ package (Mendiburu, 2017). Plant α diversity indices (iSimpson, Shannon, Simpson and Pielou’s indices), fungal community α (Chao1, AEC, Simpson, and Shannon indices) and β diversity indices were also computed (Schloss et al., 2009; Kembel et al., 2010). Additionally, to explore microbial spatial variation (β diversity), we used the Bray-Curtis metric to assess taxonomic variation. The PCoA and ANOSIM were also used to conduct community structure analyses. To characterize specialist OTUs, we computed the specificity and occupancy of each OTU across the four study sites. Specificity reflects the average abundance of an OTU within a given habitat, whereas occupancy indicates its frequency of occurrence across samples (Dufrêne and Legendre, 1997; Gweon et al., 2021). OTUs exhibiting specificity and occupancy values ≥ 0.7 were classified as specialists, indicating their habitat specificity and widespread presence (Gweon et al., 2021). Taxonomic trees were applied to illustrate the distribution and composition of specialist species across the sites by the ‘metacoder’ package (Foster et al., 2017). To determine the vegetative and edaphic effects on fungal community diversity and structure, we performed a Mantel test (permutations 999) using the ‘vegan’ package (Sunagawa et al., 2015). Fungal community composition was assessed using Bray-Curtis distances, while environmental dissimilarities were calculated based on Euclidean distances. Additionally, the phylogenetic-bin-based null model analysis (iCAMP) was employed to analyze fungal community assembly processes (Ning et al., 2020). This framework integrates the β-net relatedness index and the Raup-Crick index to evaluate phylogenetic and taxonomic beta diversity, respectively (Stegen et al., 2013; Ning et al., 2020). The relative contributions of five assembly processes, i.e., the heterogeneous selection, homogeneous selection, dispersal limitation, homogenizing dispersal, and drift were quantified, and their variations across the four sites were compared using the Wilcoxon test (Freire-Zapata et al., 2024). More detailed descriptions of the statistical analyses can be found in Xue et al. (2025). All data were processed in R software (version 4.0.2).

## 3 Results

### 3.1 Soil fungal community diversity across different restoration stages.

**Figure 2:**
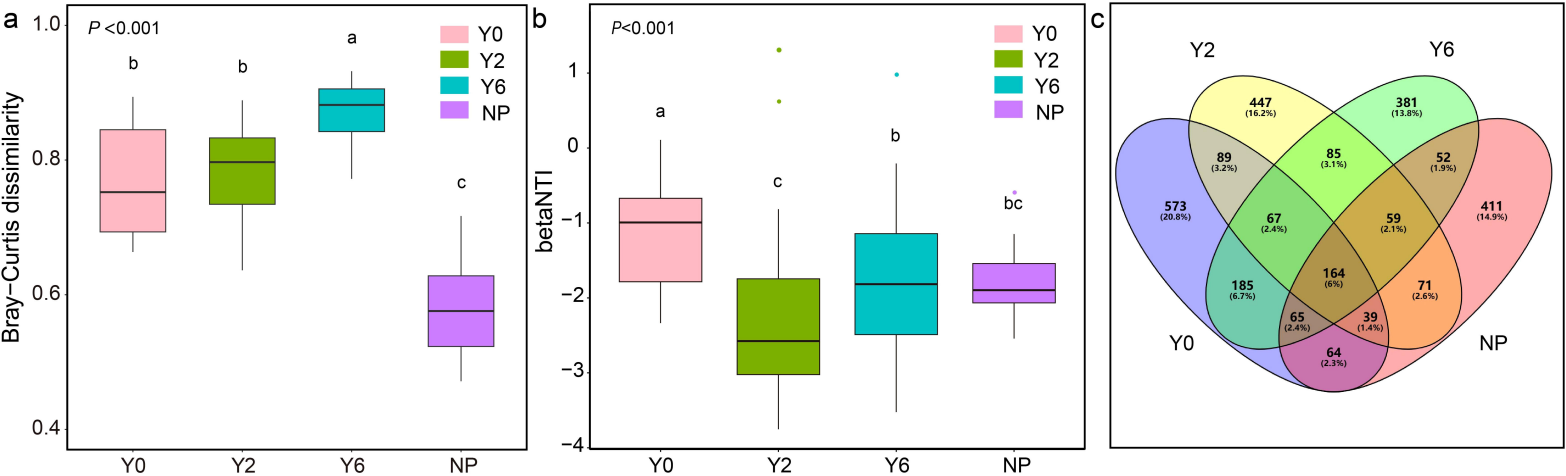
Comparisons of fungal community Bray-Curtis dissimilarity **(a)**, betaNTI **(b)**, and OTUs **(c)** among different sampling sites.

Following 2 years of artificial restoration, the Shannon and Simpson indices exhibited a significant increase (**Table 1**), whereas β diversity significantly decreased (**Figure 2B**). As the duration of restoration progressed, both the richness (Chao1 and ACE indices) and diversity (Shannon and Simpson indices) of the soil fungal communities showed a significant decline, while β diversity experienced a notable increase. Furthermore, no significant differences were detected in neither α diversity nor β diversity of the soil fungal communities between natural plant sites and the 6-year restoration sites. Multivariate statistical analysis, based on Bray-Curtis dissimilarity matrices, revealed significant differences in taxonomic compositions across the four study sites (**Figure 2A**). Specifically, only 167 OTUs were shared among all four sites, accounting for 6% of the total OTUs, with the unrestored sites, 2-year restored sites, 6-year restored sites, and natural plant sites containing 573, 447, 381, and 411 unique OTUs, respectively (**Figure 2C**).

**Table 1:** Fungal community α diversity indices among four sites.

Index	Y0	Y2	Y6	NP	*p* value
Chao1	506.07 ± 80.06 a	459.79 ± 72.01 a	340.76 ± 95.44 b	328.50 ± 67.03 b	<0.001 ***
ACE	513.77 ± 89.30 a	524.08 ± 87.36 a	359.85 ± 100.76 b	354.27 ± 45.48 b	<0.001 ***
Shannon	3.33 ± 0.58 b	4.21 ± 0.29 a	3.79 ± 0.35 ab	3.36 ± 0.51 b	0.004 **
Simpson	0.11 ± 0.10 a	0.04 ± 0.02 b	0.06 ± 0.03 ab	0.09 ± 0.05 ab	0.011 *

***, **, and * indicate *p* < 0.001, *p* < 0.01, and *p* < 0.05, respectively.

### 3.2 Soil fungal community structure and assembly mechanism across different restoration stages

**Figure 3:**
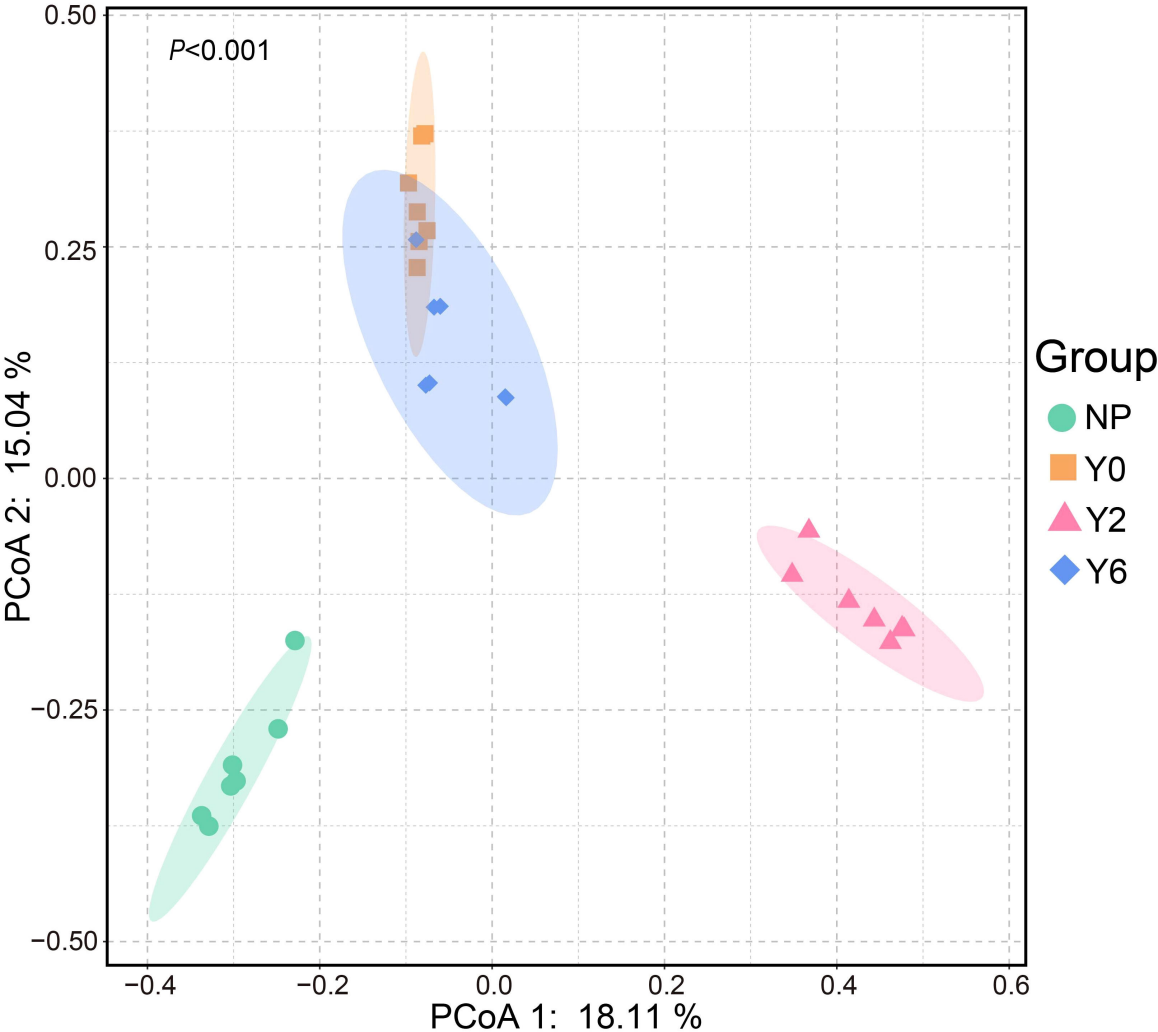
Fungal community structures under different sampling sites based on the PCoA analysis.

**Figure 4:**
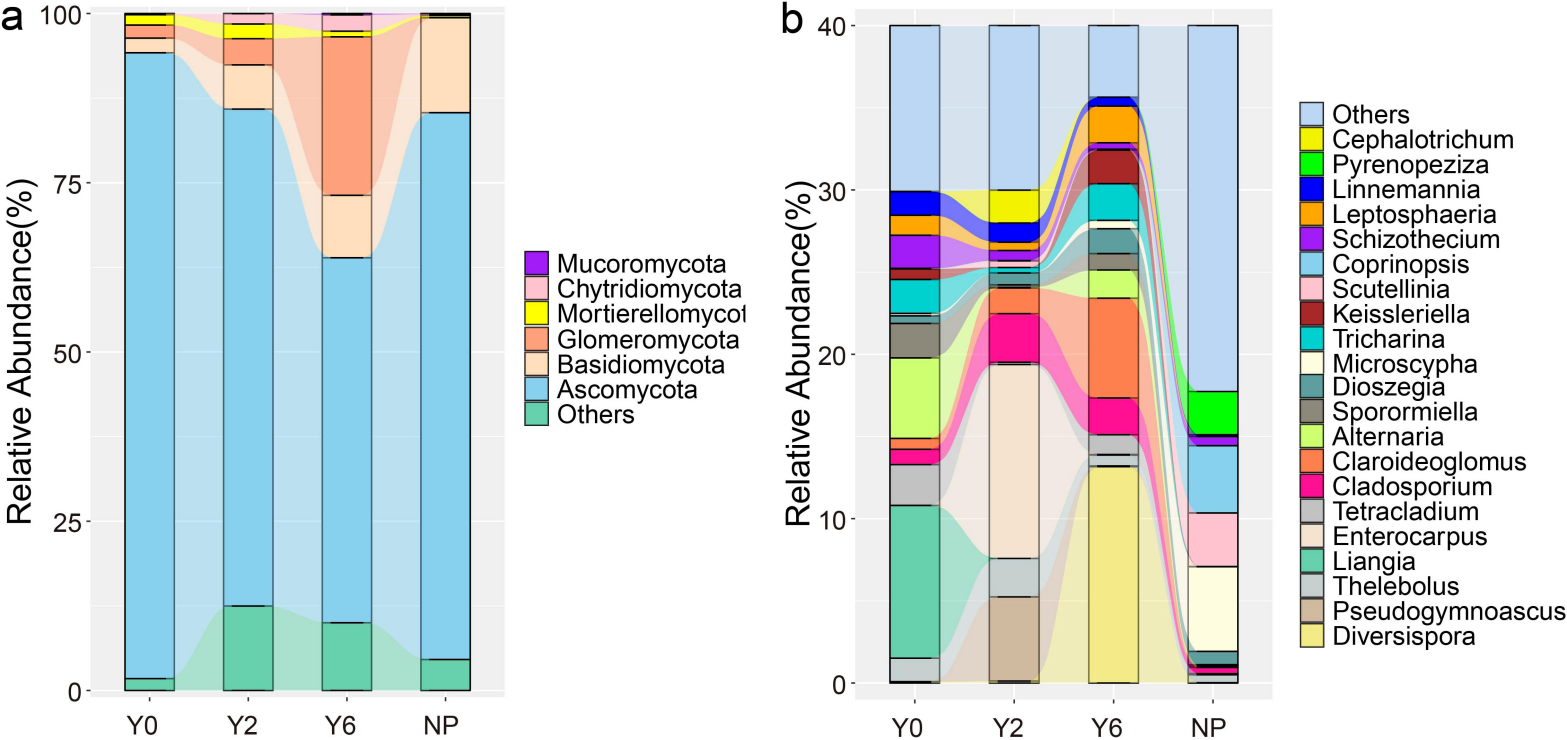
The relative abundance of soil fungi at phylum **(a)** and genus **(b)** level from four sites.

Based on PCoA, we found that artificial restoration significantly altered the composition of soil fungal communities, which was further supported by ANOSIM results (**Figure 3**). Ascomycota was the predominant phylum in all sites, exhibiting a significant decrease in relative abundance following artificial restoration (**Figure 4A**). As the years progressed, its relative abundance continued to decrease significantly. In contrast, Basidiomycota was the second dominant phylum in the unrestored, 2-year restoration, and natural plant sites, with a notable increase in relative abundance in the restoration sites. Glomeromycota was the third dominant phylum in the unrestored and 2-year restoration sites and the second dominant phylum in the 6-year restoration sites, showing a significant increase in relative abundance after 6 years of restoration.

Our study revealed that the dominant fungal genera varied significantly across different sites (**Figure 4B**). In the unrestored sites, *Liangia* and *Flavocillium* were the dominant genera. After 2 years of artificial restoration, the dominant genera shifted to *Enterocarpus* and *Pseudogymnoascus*. As the restoration period progressed, *Diversispora*, *Coniochaeta*, and *Claroideoglomus* became the dominant genera. In natural plant sites, *Boudiera* and *Microscypha* were the dominant genera. Overall, following artificial restoration, the relative abundance of *Liangia* and *Flavocillium* decreased significantly, with the highest abundance found in natural plant sites. In contrast, the abundance of *Enterocarpus* and *Pseudogymnoascus* increased significantly in the 2-year restoration sites but decreased as the restoration period continued. Their relative abundance was lowest in natural plant sites, suggesting a positive response to long-term artificial restoration. After 6 years of restoration, the abundance of *Diversispora*, *Coniochaeta*, and *Claroideoglomus* increased significantly.

**Figure 5:**
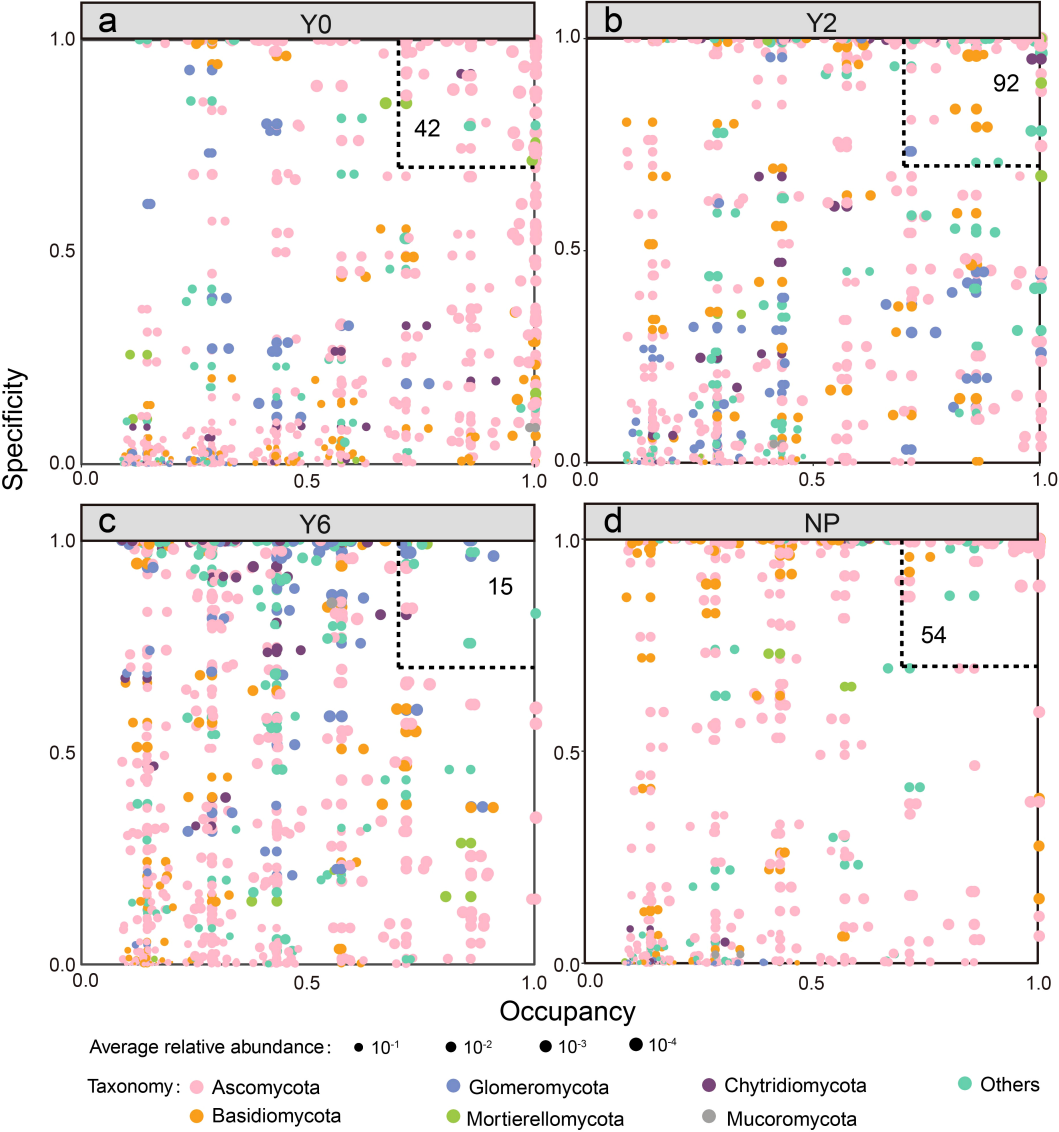
The specificity occupancy plot of soil fungal communities in Y0 unrestored sites **(a)**, Y2 2-year artificial restoration sites **(b)**, Y6 6-year artificial restoration sites **(c)**, and NP natural plant sites **(d)**.

**Figure 6:**
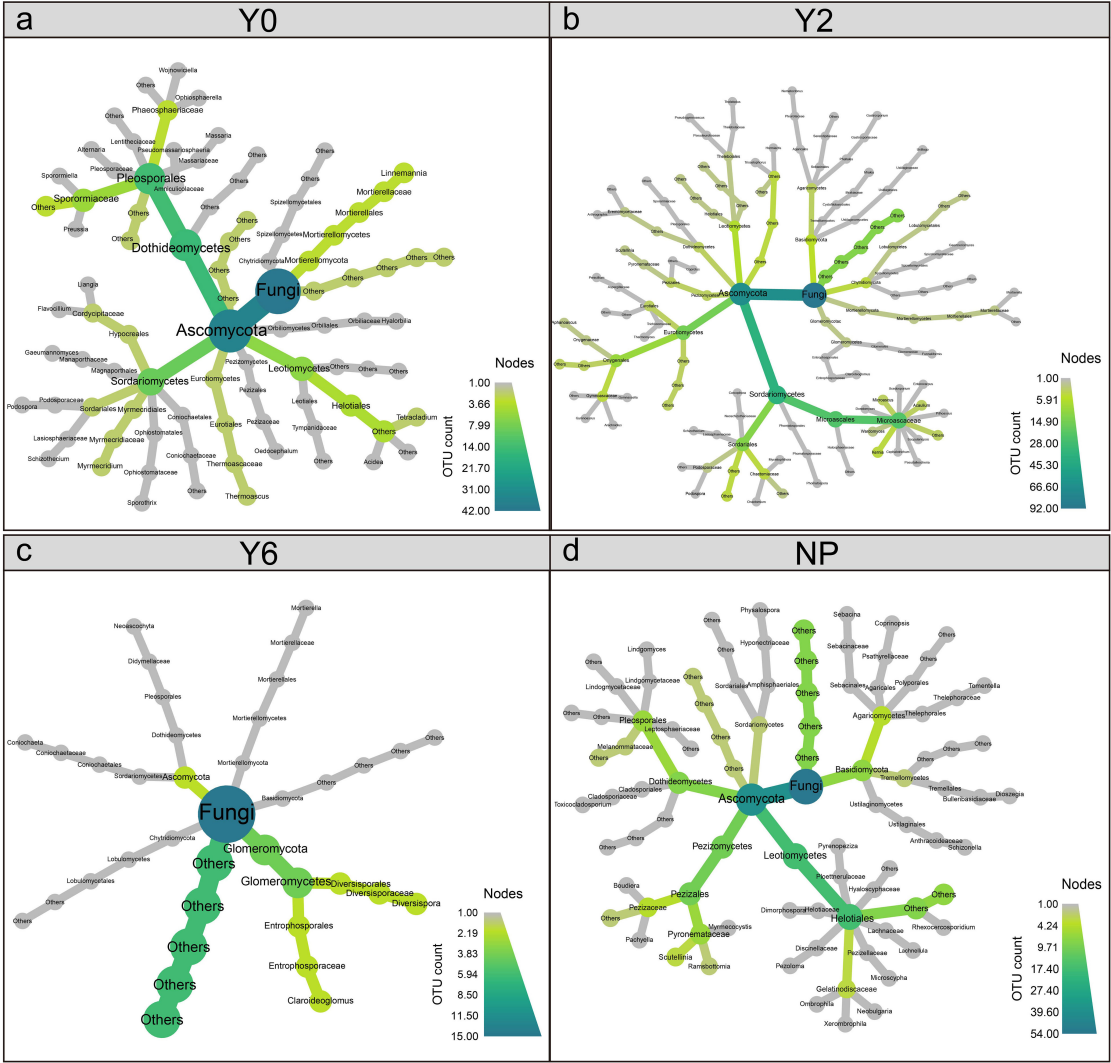
The taxonomic trees plotted of soil fungal communities in Y0 unrestored sites **(a)**, Y2 2-year artificial restoration sites **(b)**, Y6 6-year artificial restoration sites **(c)**, and NP natural plant sites **(d)**.

The occupancy and specificity analysis showed that numbers of specialist species in unrestored, 2-year restoration sites, 6-year restoration sites, and natural plant sites were 42, 92, 15, and 54, respectively (**Figure 5**). Specialist species in the unrestored, 2-year artificial restoration, and natural plant sites were mostly Ascomycota, while Glomeromycota was the predominant group of specialist species in the 6-year artificial restoration sites (**Figure 6**).

**Figure 7:**
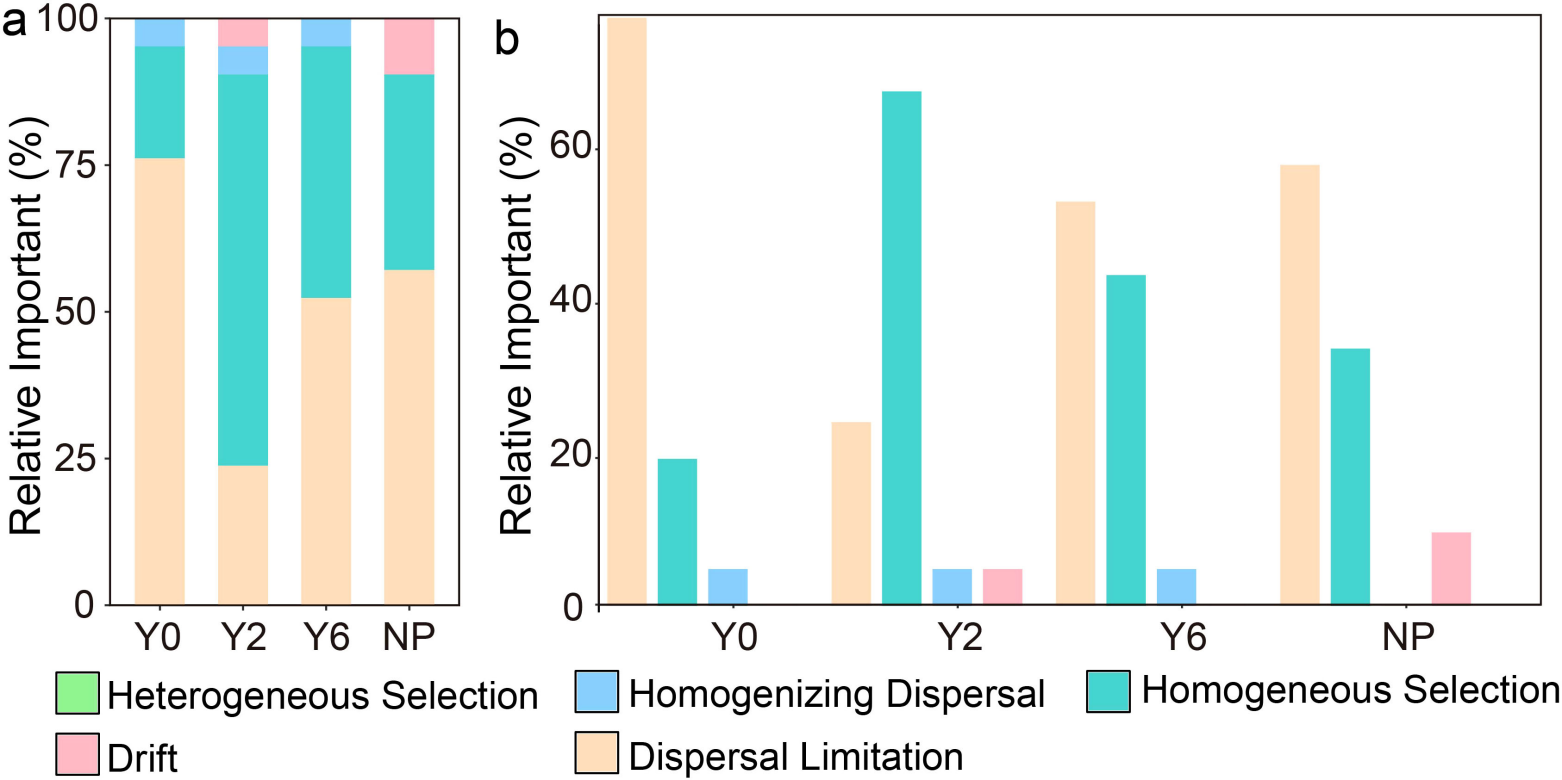
Stacked **(a)** and bar **(b)** chart of the relative contribution of each ecological process driving fungal community assembly within four sites based on null model analysis.

In **Figure 7**, we found that dispersal limitation and homogeneity selection predominantly governed the soil fungal community in alpine mining areas, but their relative importance varied as the duration of artificial restoration increased. Specifically, dispersal limitation had the highest relative importance in the unrestored and natural plant sites, followed by homogeneity selection. In the wake of artificial restoration, it was discerned that the primary driver shaping fungal communities was homogeneity selection, with dispersal limitation exerting a secondary influence. After artificial restoration, homogeneity selection was identified as making the greatest contribution to structuring fungal communities, followed by dispersal limitation. As the restoration period progressed, the relative importance of homogeneity selection decreased, while that of dispersal limitation increased, thus both homogeneity selection and dispersal limitation jointly dominated the structure of the soil fungal community in the 6-year restoration sites.

**Figure 8:**
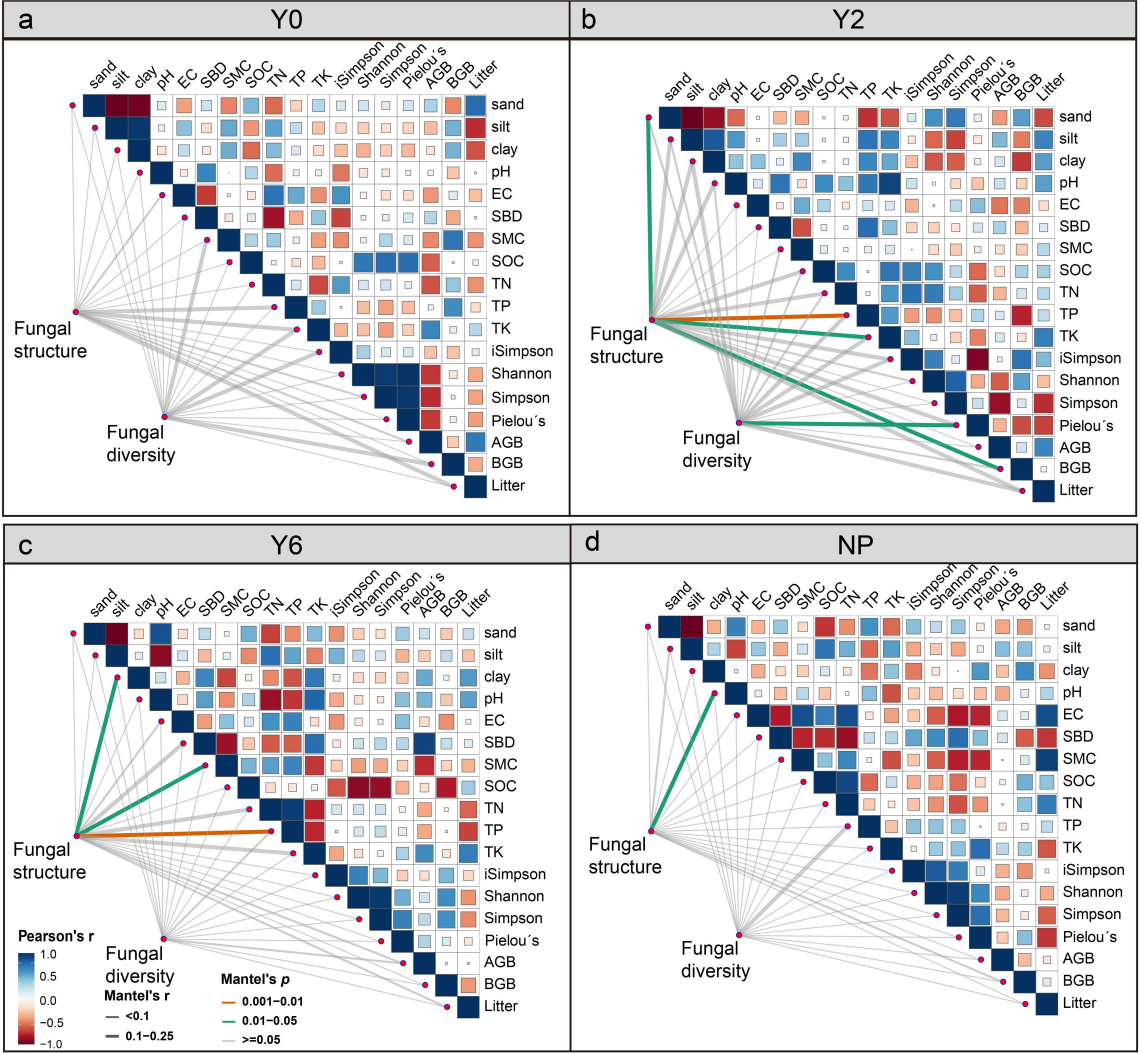
Associations of the fungal community structure with environmental factors in Y0 unrestored sites **(a)**, Y2 2-year artificial restoration sites **(b)**, Y6 6-year artificial restoration sites **(c)**, and NP natural plant sites **(d)**.

We further investigated the influence of environmental factors (**Supplementary Table S1**) on soil fungal communities in alpine mining region. The results showed that in unrestored sites, soil fungal community structure and diversity were not significantly influenced by soil and vegetation properties (**Figure 8A**). However, in 2-year artificial restoration sites, soil fungal community structure was mainly correlated with soil sand, TP, TK, and BGB (**Figure 8B**). Meanwhile, the diversity of fungal community was significantly influenced by plant Pielou’s evenness. As the restoration period progressed, the structure of fungal community was significantly affected by soil clay, SMC, and TP (**Figure 8C**). While in the natural plant sites, soil pH was the primary factor influencing soil fungal community structure (**Figure 8D**).

## 4 Discussion

### 4.1 Soil fungal community diversity across different restoration stages

We employed α and β diversity to assess fungal community diversity. Our results revealed a significant increase in the diversity indices pertaining to soil fungal communities. This may be attributed to the increased plant community richness following 2 years artificial restoration, which promotes the release of various root exudates with binding properties, thereby providing more ecological niches for fungal communities and increasing the α diversity of the fungal community (Butler et al., 2003; Dey et al., 2012; Xu et al., 2022). The restoration process may have also been affected by the addition of sheep dung, which contributed to a swift and significant enhancement of soil nutrient levels, resulting in a more balanced species composition by replacing the dominant species with other species and reducing the number of dominant species in the community (Yang et al., 2024). However, after 2 years of restoration, soil heterogeneity decreased, and the more homogeneous environment selectively filtered species in a similar way, leading to a reduction in β diversity (Nemergut et al., 2013). The fungal community α diversity in 6-year restoration sites was lower than that in 2-year restoration sites. This is probably associated with the depletion of soil nutrients as the intensity of post-restoration management and maintenance has gradually diminished during the later stages of vegetation restoration. While the β diversity of the fungal community may depend on dispersal processes in the 6-year restoration sites (Nemergut et al., 2013; Ernakovich et al., 2022), where the proportion of homogeneous selection significantly decreased and the proportion of dispersal limitation significantly increased, dispersal limitation can increase β diversity by causing spatial aggregation of species (Zhang et al., 2008). Our study observed that fungal community richness was greatest in unrestored soils, potentially due to the extremely low soil nutrient levels, which hinder plant growth. In a barren environment, plants tend to adopt a conservative approach by generating leaf litter abundant in phenolic compounds. Since soil bacteria are less proficient at decomposing such litter, fungi become more dominant in the microbial community, leading to a rise in fungal diversity (Wright et al., 2004), and our findings provide new insights into the mechanisms by which bacteria and fungi are influenced by plants in nutrient-poor soils, offering valuable perspectives for future research. Furthermore, the poor nutrient availability in the unrestored soils restricted the spread of soil fungi, leading to higher β diversity in these soils compared to other sites.

### 4.2 Soil fungal community structure and assembly mechanism across different restoration stages

Through modifications to the vegetation community composition and soil conditions, artificial restoration has markedly altered the composition and structure of the soil fungal community (Yuan et al., 2021). Ascomycota, being the most abundant and widely distributed fungal group in soil (Al-Sadi et al., 2017; Egidi et al., 2019), possesses dark-colored hyphae and large, multicellular black spores that enable it to endure extreme environmental conditions (Sterflinger, 2006; Bates et al., 2010). Hence, it is unsurprising that Ascomycota dominates across all the study sites. Following 2 years of artificial restoration, the relative abundance of Ascomycota showed a significant decrease, which may be attributed to the elevated total phosphorus levels resulting from the restoration process (Yang et al., 2024). Furthermore, as the restoration period extended, the significant increase in soil pH contributed to the decline in its relative abundance (Zhang Z, et al., 2022).

Following 2 years of restoration, the relative abundance of the *Pseudogymnoascus* within the Ascomycota significantly increased. These species has the capacity to withstand cold stress by allocating nutrients towards the mending and restoration of cellular damage, concurrently reducing the synthesis of proteins necessary for cellular expansion (Bakar et al., 2024). Thus, it is typically found in cold environments such as the Arctic, alpine, and Antarctic regions (Blehert et al., 2009; Vanderwolf et al., 2013; Wilson et al., 2017; Chaturvedi et al., 2018). Previous study indicated that the relative abundance of *Pseudogymnoascus* showed strong positive correlation with soil carbon, nitrogen and phosphorus. The substantial increase in soil nutrient content following 2 years of restoration contributed to a marked rise in its relative abundance. However, after 6 years of restoration, as soil nutrient content significant declined, the relative abundance of *Pseudogymnoascus* correspondingly notably decreased. Additionally, *Pseudogymnoascus* was positively correlated with soil bulk density, thus, the lower soil bulk density will limit the growth of *Pseudogymnoascus* in the unrestored sites. *Coniochaeta* is also a member of Ascomycota, and it engages with plant life through the release of proteins that initiate symbiotic relationships (Challacombe et al., 2019). Its relative abundance significantly increased in the 6-year restoration sites compared to the other three sites, which may be associated with the variety of plant species present.

Members of the Basidiomycota primarily rely on exogenous materials, such as plant litter or soil organic matter, as their main carbon sources for growth and reproduction. They significantly affect the degradation of plant residues, especially the breakdown of recalcitrant macromolecular organic compounds, through the production of oxidative and hydrolytic extracellular enzymes (Entwistle et al., 2018). In contrast to unrestored areas, the artificial restoration sites exhibited a notable rise in plant residues, which correlated with an elevated relative abundance of Basidiomycota. Additionally, Basidiomycota, functioning as saprotrophic fungi, possess the capability to break down lignin and cellulose, even in anaerobic environments (Guo et al., 2018; Kui et al., 2021), which likely explains their highest relative abundance in the natural plant sites. *Microscypha*, one of the major members of Basidiomycota, has been confirmed to possess plant growth-promoting and biocontrol functions (Gao et al., 2023) and is a core species in the root systems and rhizosphere soils of *Leymus chinensis* in alpine meadow (Guo et al., 2024). Our findings also indicated that its relative abundance is highest in the natural plant sites.

Glomeromycota are predominantly composed of arbuscular mycorrhizal fungi, which can establish mutualistic symbioses with the roots of 72% of higher flowering plants (Meng et al., 2023), playing an essential role in plant nutrient uptake and growth (Choi et al., 2018). In comparison to the unaltered areas, the plant community richness in the artificially restored regions increased significantly, resulting in increasing relative abundance of Glomeromycota. It even became the second most dominant group in the 6-year restoration sites, with *Diversispora* and *Claroideoglomus* emerging as the dominant genera in the same sites. However, our findings also indicated that, while the plant community richness was greatest in the natural plant sites, the relative abundance of Glomeromycota was comparatively low. This could be due to the pronounced host specificity inherent in the symbiotic association between Glomeromycota and plants (Liu et al., 2012; Zhang et al., 2023).

Artificial restoration impacts not only the composition of dominant species in soil fungal communities but also the processes that govern community assembly. In unrestored sites, the poor soil quality and sparse vegetation restrict fungal growth and reproduction, meaning the community structure is primarily shaped by dispersal limitations, with less influence from environmental factors such as vegetation and soil. In the 2-year restoration sites, significant increases in soil nutrient content, plant diversity, and biomass resulted in soil fungal community structure primarily govern by homogenous selection, with soil and vegetation becoming the primary influencing factors. Mate analysis further revealed that the soil fungal community structure was chiefly determined by soil sand, TP, TK, and BGB. In 6-year restoration sites, decreased soil nutrient content and greater heterogeneity resulted in both homogenous selection and dispersal limitations jointly influencing fungal community structure. Here, the structure was mainly driven by soil clay, SMC, and TP. In natural plant sites, higher BD and SMC impaired soil aeration, thus, the fungal community is primarily influenced by dispersal limitation.

### 4.3 Limitations and future directions

The current analysis only focuses on the diversity and assembly mechanisms of fungal communities, without integrating the functional aspects of these communities to reveal the dynamic changes in key functional genes. Future research should combine functional gene analysis to clarify the direct relationship between fungal functional activity and ecosystem functioning. This study only collected samples from a single location in the Muli mining area, and future studies should expand the sampling to multiple regions to explore how the relative contributions of environmental filtering and dispersal limitation change across spatial scales. Additionally, this study only collected samples from sites with 2 and 6 years of artificial restoration, which makes it difficult to accurately investigate the successional characteristics of soil fungal community structure following artificial restoration. Future studies should include longer temporal sequences to more precisely quantify the impact of human interventions on fungal communities.

## 5 Conclusion

The implementation of artificial restoration measures has substantially augmented the diversity indices of soil fungal communities, while simultaneously causing a notable decline in β diversity in alpine mining areas. As the restoration period extended, the α diversity of soil fungal communities significantly decreased, while β diversity significantly increased. Concurrently, artificial restoration induced significant structural modifications in the soil fungal community composition, with distinctive fungal species accounting for over 82% of the total community in restored sites. Additionally, we found that artificial restoration had a positive effect on Basidiomycota. Dispersal limitation and homogeneity selection were the dominant process driving soil fungal community assembly in alpine mining areas. In the natural plant and unrestored sites, the structure of soil fungal communities was mainly influenced by dispersal limitation, followed by homogeneous selection. After artificial restoration, the structure of soil fungal community primarily was governed by homogeneous selection, following the dispersal limitation. As restoration time progressed, the importance of homogeneous selection decreased, while the relative importance of dispersal limitation notably increased, and the structure of soil fungal community mainly influenced by soil clay, SMC, and TP.

## Data Availability

The original contributions presented in the study are publicly available. This data can be found here: https://www.ncbi.nlm.nih.gov/bioproject/PRJNA1208394/.
