# Supplementary material for: Effects of vegetation restoration on soil fungi community structure and assembly process in a semiarid alpine mining region
Source: Front Plant Sci. 2025 Jul 4;16:1579142. doi: 10.3389/fpls.2025.1579142 (PMC12272346; doi:10.3389/fpls.2025.1579142)
Supplement: Supplementary file 1 [file Table1.docx]

Table S1 The ANOVA results of soil and plant properties among different sites

| **soil properties** | **Y0** | **Y2** | **Y6** | **NP** | ***p* value** |
| --- | --- | --- | --- | --- | --- |
| sand | 32.98+11.28 a | 32.34+7.17 a | 43.37+7.87 a | 34.53+8.30 a | 0.100 |
| silt | 62.84+10.21 a | 65.67+7.09 a | 54.06+7.69 a | 63.64+8.02 a | 0.07 |
| clay | 4.08+1.16 a | 1.98+0.56 b | 2.57+0.54 b | 1.58+0.40 b | <0.001 |
| SBD | 1.78+0.15 a | 1.01+0.33 b | 1.74+0.22 a | 0.41+0.09 c | <0.001 |
| SMC | 10.60+3.59 c | 33.18+9.56 b | 16.13+4.67 c | 66.81+5.36 a | <0.001 |
| EC | 80.94+20.01 b | 181.77+35.34 a | 83.54+40.24 b | 60.19+7.37 b | <0.001 |
| pH | 8.69+0.29 a | 8.17+0.16 b | 8.68+0.20 a | 6.43+0.15 c | <0.001 |
| SOC | 51.60+20.10 b | 136.61+26.06 a | 45.55+19.78 b | 106.73+14.39 a | <0.001 |
| TN | 1.80+0.56 b | 11.22+2.678 a | 2.09+0.61 b | 12.20+2.19a | <0.001 |
| TP | 0.50+0.15 c | 1.34+0.26 a | 0.47+0.04 c | 0.83+0.123 b | <0.001 |
| TK | 31.62+3.63 a | 27.32+2.15 ab | 31.48+1.82 a | 24.32+3.81 b | <0.001 |
| **Plant properties** |  |  |  |  |  |
| AGB | 2.39+1.26 c | 156.56+46.95 a | 28.55+12.16 c | 88.03+20.91 b | <0.001 |
| BGB | 35.69+8.86 c | 1469.77+310.81 b | 179.06+48.71 c | 4741.89+1608.84 a | <0.001 |
| Litter | 13.23+4.52 b | 148.98+22.40 a | 3.55+2.18 b | 0.97+0.20 b | <0.001 |
| Isimpson | 2.14+0.378 b | 3.86+1.68 b | 3.86+1.21 b | 10.57+0.98 a | <0.001 |
| Shannon | 0.67+0.04 b | 0.63+0.25 b | 0.93+0.39 ab | 1.17+0.29 a | 0.004 |
| Simpson | 0.47+0.03 a | 0.37+0.16 a | 0.52+0.18 a | 0.53+0.15 a | 0.144 |
| Piekou | 0.92+0.12 a | 0.51+0.20 b | 0.71+0.21 ab | 0.50+0.12 b | <0.001 |

Note: Y0: unrestored sites; Y2: 2-year artificial restoration sites; Y6: 6-year artificial restoration sites; NP: natural plant sites.
